# Circulation of oxytetracycline- and ciprofloxacin-resistant commensal *Escherichia coli* strains in broiler chickens and farm environments, Bangladesh

**DOI:** 10.14202/vetworld.2020.2395-2400

**Published:** 2020-11-10

**Authors:** Avijit Das, Pangkaj Kumar Dhar, Avijit Dutta, Mohammad Shah Jalal, Priya Ghosh, Tridip Das, Himel Barua, Paritosh Kumar Biswas

**Affiliations:** Department of Microbiology and Veterinary Public Health, Chattogram Veterinary and Animal Sciences University, Khulshi, Chattogram, Bangladesh

**Keywords:** antimicrobial resistance, *Escherichia coli*, farm environment, poultry

## Abstract

**Background and Aim:**

The emergence of antimicrobial resistance (AMR) in commensal organism, such as *Escherichia coli* of food animals, is an alarming issue for global health. It increases the possibility of transmitting AMR determinant(s) to human bacterial pathogens by transferable genetic materials, particularly by plasmids. Hence, it is important to know which resistant genes are being carried by commensal organisms in food chain in a country and their level of temporal loads. As a result, pre-emptive measures can be advocated with an aim to reduce their risks in their primary source of circulation which consequently would benefit the public health.

**Materials and Methods:**

Commensal *E. coli* strains from broiler chickens on randomly selected 30 farms and the farm environments were examined for the frequencies of isolation of resistant strains to oxytetracycline and ciprofloxacin. Five birds were randomly selected from each farm to collect cloacal swab samples (total of 150 samples). Furthermore, a total of 150 environmental samples comprising one each from feed, water, soil, litter, and litter damping site of each farm were screened for the isolation of commensal *E. coli* strains. Strains thus obtained were initially tested for their resistance to oxytetracycline and ciprofloxacin by Kirby–Bauer disk diffusion method. Oxytetracycline-resistant strains were further screened for the presence of resistance determining genes, namely, *tetA*, *tetB*, and *tetC* by uniplex polymerase chain reactions. Risks associated with the isolation frequency of oxytetracycline- and ciprofloxacin-resistant *E. coli* were also assessed by univariable logistic regression analysis.

**Results:**

The results revealed that all *E. coli* isolates, regardless of the source of origin, were resistant to oxytetracycline, while 78.4% (95% confidence interval [CI] 69.1-85.5%) showed resistance to ciprofloxacin. All the randomly selected (20) oxytetracycline-resistant strains harbored the *tetA* gene, whereas *tetB* and *tetC* were reported in three and two isolates, respectively. After univariable analysis, only one variable, that is, strain 1 of broiler chickens compared to two other strains was found to be positively associated with the isolation of ciprofloxacin-resistant *E. coli* (odds ratio 12.75 [95% CI 1.0-157.1], p=0.047).

**Conclusion:**

Resistance emerged against oxytetracycline and ciprofloxacin in commensal *E. coli* strains circulating in live poultry and farm environments in Bangladesh seems to be very high. Thus, human infection with drug-resistant *E. coli* strains through food chain will critically compromise the therapeutic measures currently available.

## Introduction

Antimicrobial resistance (AMR) is now considered as one of the three greatest threats to public health globally. Antibiotic-resistant organism has the potential to affect any species (both animal and human), of any age, in any country [1]. One of the main causes of the emergence of AMR in both pathogenic and commensal bacteria is the indiscriminate use of antibiotics in food animals. In Bangladesh, antimicrobials are being widely used in food animals, particularly in poultry for prophylaxis and therapeutic purposes [2]. Some poultry farmers consider that using one or more antibiotics at different ages of birds is a routine program of poultry rearing and they do so. These unnecessary usages of antibiotics might have roles in the emergence of AMR in commensal bacteria, such as *Escherichia coli*, of poultry. This emergence of drug resistance in commensal *E. coli* of poultry is an important issue to describe as they can be easily transmitted to human, through food chain, as poultry is the easily available protein source throughout the world. Furthermore, the antimicrobial-resistant *E. coli* has the potential to transfer the AMR determinants to other closely related bacterial pathogens by transferrable genetic elements, such as plasmids.

Over the years, researchers have reported the emergence of plasmid-mediated resistance in animal origin *E. coli* against many key antimicrobials including third-generation cephalosporins [3-6] and their transmission from animals to human through food chain or husbandry practice [7,8]. For an example, poultry workers in the United States were significantly more likely to carry *E. coli* resistant to gentamycin, an antibiotic of limited human use than community controls who were not involved with poultry production [9]. Poultry farmers had a higher prevalence of carriage of ciprofloxacin-resistant *E. coli* (17%) than subjects participating in other studies (<1-3%) in the 1990s [5]. Once antimicrobial-resistant *E. coli* are available in the environment, migratory birds, wild animals, and invertebrates, they may further contribute to the dispersal of AMR genes [10,11]. Such strains can also gain entrance to food and water sources along with the final poultry products or indirectly by certain practices of the farm owners, such as selling of the poultry litter as biofertilizer or disposing of poultry wastes and litter into water bodies.

The aim of the present study was designed to assess the frequency and distribution of *E. coli* strains isolated from live broiler chickens and their farm environment that had acquired resistance to oxytetracycline and ciprofloxacin. Screening for the presence of some of the predominant genes determining oxytetracycline resistance was also carried out and risks associated with the isolation frequency of oxytetracycline- and ciprofloxacin-resistant strains were further evaluated.

## Materials and Methods

### Ethical approval

All the applicable ethical guidelines for animal were followed during handling and sample collection from birds and adequate measures were taken to minimize pain or discomfort of selected birds. This project was approved by the ethical committee of Chittagong Veterinary and Animal Sciences University, Bangladesh.

### Sampling, study period and location

A cross-sectional survey was done with sampling of broiler chickens on 30 farms randomly selected from the district of Chittagong, Bangladesh, and their environments. The minimum sample size required for the birds and the farm environment each to be screened was 100 based on the formula π (1–π)/e2, where π is the prevalence and e is the standard error [12]. We presumed that the prevalence of birds or farm environment with commensal *E. coli* strain resistant to either oxytetracycline or ciprofloxacin was 0.5 with standard error of 0.05. Therefore, the minimum sample size required for each category (birds or farm environment) was 100. However, we sampled 150 live broiler chickens on the selected farms and 150 environmental samples comprising 30 each belonging to feed, water, soil, litter, and litter damping site. A two-stage approach: Random sampling of 30 farms followed by random sampling of five birds from each farm and five environmental samples from each of the selected farm was performed between January and June 2017. Cloacal swabs were taken with sterile swabs from the live birds while five swabs collected from each of the environmental sites of a farm were pooled into Stuart’s transport medium and shipped to the laboratory for bacteriological investigations.

### Isolation and identification of *E. coli*

For isolation of *E. coli* from a collected sample, the sample was at first inoculated into a test tube containing buffer peptone water (Oxoid Ltd., Basingstoke, Hampshire, UK) and incubated at 37°C overnight for primary enrichment. The overnight culture was streaked on MacConkey agar medium (Oxoid Ltd., Basingstoke, Hampshire, UK) and incubated at 37°C for 24 h. Bright pink-colored large colonies yielded on to MacConkey agar plate were suspected as the growth of *E. coli*. Such colonies were streaked onto EMB agar plate (Oxoid Ltd., Basingstoke, Hampshire, UK) and incubated at 37°C for 24 h. Based on “green metallic sheen,” colony yielded on this medium was taken as the growth of *E. coli*, which was later confirmed by applying standard biochemical tests recommended for the identification of *E. coli*.

### Screening resistance of *E. coli*

Sensitivity patterns of *E. coli* isolated to oxytetracycline and ciprofloxacin were tested using disk diffusion method according to the method described by Kirby–Bauer [13] method and the results were interpreted according to Clinical and Laboratory Standard Institute [14] guidelines.

### Detection of three tet genes in the oxytetracycline-resistant strains

Strains of *E. coli* that showed resistance to oxytetracycline were recultured on blood agar. DNA from a culture on blood agar was extracted using the conventional boiling method [15]. Specific primer sequences for amplification of *tetA, tetB*, and *tetC* were used as reported before [16]. A thermocycler (Applied Biosystems, Singapore) was used for amplification of DNA. The polymerase chain reaction products were visualized under UV transilluminator (BDA digital, Biometra GmbH, Germany) after gel electrophoresis using 1.5% agarose gel.

### Statistical analysis

The data were entered into a spreadsheet program of Microsoft Excel 2010 and transferred to STATA 11 (StataCorp, College Station, Texas, USA) for data summary and analysis. To estimate the strength and statistical significance of association of a variable with the isolation frequency of oxytetracycline- and ciprofloxacin-resistant *E. coli*, univariable logistic regression analysis was done. An association was considered significant if a variable had p <0.05.

## Results

### Prevalence of *E. coli*

Of the 150 cloacal samples investigated, 89 (59.3%; 95% confidence interval [CI] 51.3-66.9%) yielded *E. coli*. Regardless of sources, on the other hand, 38% (95% CI 30.6-46%) environmental samples tested positive for *E. coli*. Among the environmental samples, the isolation frequency of *E. coli* was higher in litter samples compared with others. An overview of the numbers of samples collected from the 30 selected broiler farms and their environments along with the isolation status of *E. coli* are shown in Table-1.

**Table-1:** An overview of total samples collected from the selected broiler farms and their environments with the positivity of *Escherichia coli.*

Name of sample	Number of sampled	Positivity (%)	95%; CI
Live bird (cloacal swab)	150	89 (59.3)	51.3-66.9
Feed	30	9 (30)	16.52-48.2
Water	30	11 (36.7)	21.81-54.55
Soil	30	10 (33.3)	19.13-51.32
Liter	30	16 (53.3)	36.14-69.77
Liter dumping site	30	11 (36.7)	21.81-54.55

CI=Confidence interval

### Antibiotic resistance

All the isolates tested showed resistance to oxytetracycline while 78.4% (95% CI 69.09-85.45%) showed resistance to ciprofloxacin. However, the strains isolated from the cloacal samples of the live birds had a higher resistance profile to ciprofloxacin compared with the strains obtained from the farm environment. *E. coli* strains as obtained from different sources and tested for their resistance profiles to oxytetracycline and ciprofloxacin are depicted in Table-2.

**Table-2:** *Escherichia coli* strains isolated from different sources and their resistant profiles against oxytetracycline and ciprofloxacin.

Source of sample	Number of strain tested	Oxytetracycline		Ciprofloxacin	
	
		Resistant isolates (%)	95% CI	Resistant isolates (%)	95% CI
Live bird	40	40 (100)	89.6-100	35 (87.5)	73.42-95.01
Feed	9	9 (100)	65.54-100	6 (66.7)	35.09-88.27
Water	11	11 (100)	69.98-100	9 (81.8)	51.15-96.01
Soil	10	10 (100)	67.91-100	3 (30)	10.33-60.77
Liter	16	16 (100)	77.31-100	14 (87.5)	62.72-97.76
Liter dumping site	11	11 (100)	69.98-100	9 (81.8)	51.15-96.01

CI=Confidence interval

### Detection of three *tet* genes (*tetA*, *tetB*, and *tetC*)

**Figure-1:**
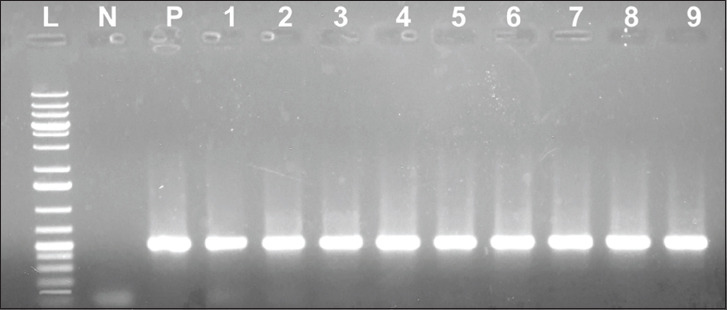
Result of polymerase chain reactions assay for the *tetA* gene of some of the isolates tested; lane L: 1 kb plus DNA ladder; lane N: Negative control; lane P: Positive control; lane 1-9: *tetA* gene sized (502 bp) amplicon.

**Figure-2:**
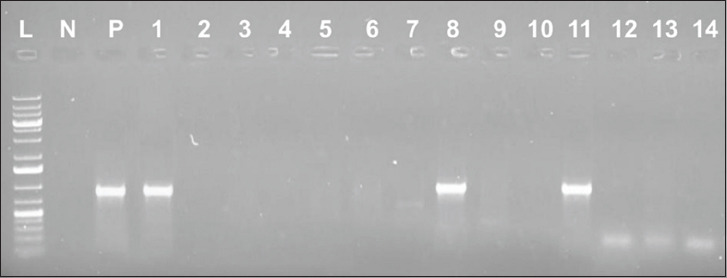
Result of polymerase chain reactions assay for the *tetB* gene of some of the isolates tested; lane L: 1 kb plus DNA ladder; lane N: Negative control; lane P: Positive control; lane 1-14: *tetB* gene sized (930 bp) amplicon.

**Figure-3:**
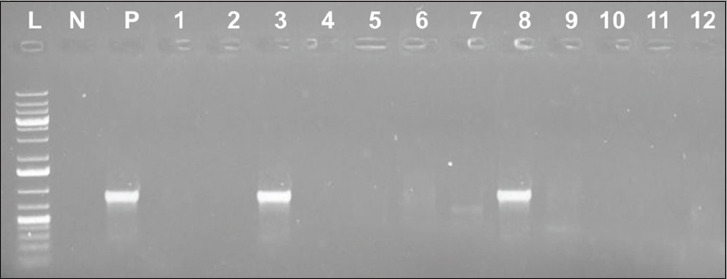
Result of polymerase chain reactions assay for the *tetC* gene of some of the isolates tested; lane L: 1 kb plus DNA ladder; lane N: Negative control; lane P: Positive control; lane 1-12: *tetC* gene sized (888 bp) amplicon.

All the 20 randomly selected *E. coli* isolates showing resistance to oxytetracycline harbored the *tetA* gene (502 bp), while three had the *tetB* gene (930 bp) and two had the *tet*C gene (888 bp) (Table-3). Isolates resistant to oxytetracycline and having the *tetA*, *tetB*, and *tetC* genes, as measured by the typical amplicon sizes of the gene products, are portrayed in Figures-1-3, respectively.

**Table-3:** Detection of *tetA*, *tetB,* and *tetC* genes in randomly selected isolates showing resistance to oxytetracycline.

Antimicrobial agent	Number of isolates	Gene tested	Positive isolates (%)	95% CI
		*TetA*	20 (100)	81.02-100
Oxytetracycline	20	*TetB*	3 (15)	4.39-36.88
		*TetC*	2 (10)	1.57-31.32

CI=Confidence interval

### Risks factors associated with *E. coli* displaying resistance to ciprofloxacin and oxytetracycline

The results of univariable analyses to assess the risks associated with the isolation frequencies of oxytetracycline- and ciprofloxacin-resistant *E. coli* are shown in Tables-4 and 5, respectively. None but only one variable, that is, broiler strain I compared with other two strains of birds screened had a higher frequency of isolation of *E. coli* possessing resistance to ciprofloxacin. However, no variables were associated with the higher or lower frequency of isolation of *E. coli* having resistance to oxytetracycline.

**Table-4:** Results of univariable analysis to assess the risks associated with the frequency of isolation of commensal *Escherichia coli* showing resistance to ciprofloxacin.

Variable	Odds ratio (95% CI)	p-value
Source of DOC		
Breeder farm 1	1.38 (0.18-10.7)	0.760
Breeder farm 2	0.17 (0.009-2.82)	0.214
Breeder farm 4	Omitted	-
Breeder farm 5	-	Reference
Strain of bird		
Strain 1	12.75 (1.0-157.140)	0.047
Strain 2	4.5 (0.25-80.577)	0.307
Strain 3	-	Reference
Age		
1^st^ week	-	Reference
2^nd^ week	1.7 (0.07-37.7)	0.748
3^rd^ week	3 (0.14-64.26)	0.482
4^th^ week	3 (0.12-73.64)	0.501
Use of antibiotics in the 1^st^ week	0.40 (0.04-4.02)	0.431
Use of antibiotics in the 2^nd^ week	1.14 (0.23-5.46)	0.873
Use of antibiotics in the 3^rd^ week	0.94 (0.194-4.52)	0.936
Use of antibiotics in the 4^th^ week	1.4 (0.22-8.76)	0.719
Commercial feed from		
Producer 1	-	Reference
Producer 2	1.1(0.142-8.67)	0.920
Producer 3	0.11(0.006-1.77)	0.120
Producer 4	1 (0.06-15.98)	1.00
Litter used for		
Aquaculture	-	Reference
Crop agriculture	0.29 (0.02-3.67)	0.342
Liquid waste from farm	-	
Drained to open farm land	0.3(0.03-4.19)	0.395
Collected by other people	0.2(0.017-2.26)	0.194
Drained to sewerage system	-	Omitted
Drained to pond or for other use	-	Reference

**Table-5:** Results of univariable analysis to assess the risks associated with the frequency of isolation of commensal ciprofloxacin-resistant *Escherichia coli* showing resistance to oxytetracycline.

Variable	Odds ratio (95% CI)	p-value
Source of DOC		
Breeder farm 1	2.99 (0.14-32.53)	0.570
Breeder farm 2	-	Omitted
Breeder farm 4	-	Omitted
Breeder farm 5	-	Reference
Strain of bird		
Strain 1	3.17 (0.21-46.73)	0.401
Strain 2	-	Omitted
Strain 3	-	Reference
Age		
1^st^ week	-	Reference
2^nd^ week	1.0 (0.05-19.34)	1.00
3^rd^ week	1.57 (0.08-29.40)	0.762
4^th^ week	-	Omitted
Use of antibiotics in the 1^st^ week	-	-
Use of antibiotics in the 2^nd^ week	0.4 (0.03-4.96)	0.476
Use of antibiotics in the 3^rd^ week	0.34 (0.03-4.27)	0.406
Use of antibiotics in the 4^th^ week	0.7 (0.05-8.97)	0.784
Commercial feed from		
Producer 1	-	Reference
Producer 2	1.83 (0.12-27.80)	0.662
Producer 3	-	Omitted
Producer 4	-	Omitted
Litter used for		
Aquaculture	-	Reference
Crop agriculture	0.29 (0.02-3.67)	0.342
Liquid waste from farm		
Drained to open farm land	1.78 (0.13-23.52)	0.662
Collected by other people	-	Omitted
Drained to sewerage system	-	Omitted
Drained to pond or for other use	-	Reference

CI=Confidence interval

## Discussion

Little is known on the circulation of commensal *E. coli* strains that have acquired resistance to commonly used antimicrobials in poultry in Bangladesh. The results of the survey revealed that all the strains as obtained from the survey were resistant to oxytetracycline. The isolation frequencies of the strains resistant to ciprofloxacin from live broilers, feed, and water used for them and used litter were also alarmingly high. These commensal *E. coli* strains can pass on their resistance traits vertically and horizontally by transferable genetic materials to other enteric pathogens, such as *Salmonella* [17]. Because ciprofloxacin is one of the critically important antimicrobials to treat enteric fever and other infections in humans in Bangladesh, circulation of *E. coli* strains resistant to this antimicrobial might have a significant public health impact indirectly [18].

In this study, all the isolates showed resistance against oxytetracycline (100%). The result is agreed with a previous study finding, where most of the poultry *E. coli* isolates were found to be resistant to oxytetracycline [19,20]. We found a high resistance pattern of *E. coli* isolates against ciprofloxacin (78.45%), which is, however, not corroborated with the previous findings [21]. This increased resistance to ciprofloxacin over time might be due to sustained and overuse of ciprofloxacin in poultry industry in Bangladesh [20,22].

None of the variables assessed in the study had any independent association with the isolation frequency of commensal *E. coli* acquiring resistance to either oxytetracycline or ciprofloxacin, although after univariable analysis, the frequency of the isolation of *E. coli* resistant to ciprofloxacin from one of the strains of the birds was significantly higher compared to two others. Therefore, again, sustained overuse or misuse of ciprofloxacin in this strain could not be ruled out for the emergence of resistant strains to ciprofloxacin and their wider circulation.

Tetracycline is a broad-spectrum antibiotic that prevents protein synthesis of bacteria by preventing aminoacyl-tRNA from binding to the ribosome. Resistance to the antibiotic is conferred by one or more of the 36 reported *tet* genes, which encode one of three mechanisms of resistance: An efflux pump, a method of ribosomal protection, or direct enzymatic inactivation of the drug [23]. Efflux mechanisms appear to be more abundant among Gram-negative bacteria, while ribosomal protection mechanisms are more common among Gram-positive. The rapid spread of tetracycline resistance among bacteria is due to the localization of *tet* genes on transferrable genetic elements such as plasmids, transposons, and integrons [24].

The acquisition of *tetA* gene in all of *E. coli* isolates showing resistance to oxytetracycline suggests that this gene is predominantly responsible for oxytetracycline resistance and it might has been transferred from the resistant strain to the susceptible one [25]. The contributions of other two genes, *tet*B and *tet*C, might be little. However, due to resource limitation, the presence of other *tet* genes in the resistant straits was not investigated.

## Conclusion

The resistance to oxytetracycline in commensal *E. coli* strains circulating in live broiler chickens and their farm environments is probably almost cent percent. Most oxytetracycline-resistant *E. coli* strains harbor the *tetA* gene, and resistance in these isolates to oxytetracycline is probably due to acquiring the *tetA* gene. Not all but more than 50% *E. coli* strains circulating in broiler chickens and some key farm environments, such as feed, water, and litter, are also resistant to ciprofloxacin. Mutations in the *GyrA* and *GyrB* genes could be the molecular mechanisms for acquiring the ciprofloxacin resistance. The isolation frequency of ciprofloxacin-resistant *E. coli* strains in the strains of broiler chickens reared is not the same.

## Authors’ Contributions

AD conceived and designed the work and conducted the fieldwork (sample collection, preservation, and transportation) with PKD, ADu, MSJ, PG, and TD. HB and PKB supervised the study. AD, PKD, ADu, and MSJ performed laboratory work, statistical analysis, interpreted the data, and drafted the manuscript. HB and PKB edited and reviewed the manuscript. All authors read and approved the final manuscript.
